# Selection of High-Performance Sorbent for H_2_S Removal and Regulation of Reaction Products via Thermodynamic Simulation

**DOI:** 10.3390/ma18122918

**Published:** 2025-06-19

**Authors:** Yanni Xuan, Shuaicheng Peng, Hong Tian, Zhangmao Hu, Yanshan Yin, Haitao Gao

**Affiliations:** 1 School of Energy and Power Engineering, Changsha University of Science and Technology, Changsha 410114, China; xuanyanni@csust.edu.cn (Y.X.); z17369231750@163.com (S.P.); huzhangmao@163.com (Z.H.); yanshan.yin@csust.edu.cn (Y.Y.); 2 State Key Laboratory of Precision Manufacturing for Extreme Service Performance, Central South University, Changsha 410083, China

**Keywords:** H_2_S removal, thermodynamic simulation, Gibbs free energy variation, metal oxide, reaction products regulation

## Abstract

Thermodynamic simulations of the H_2_S removal from blast furnace gas by metal oxides were conducted to select a suitable metal desulfurizer. Notably, the Mn oxides demonstrated themselves as the optimal H_2_S removal agents. They are characterized by the absence of radioactive pollution, high cost-effectiveness, high sulfur fixation potential, and non-reactivity with CO_2_, CO, and CH_4_. Through a comprehensive comparison of Mn oxides, the sulfur fixation potential and sulfur capacity were elucidated as follows: Mn_3_O_4_ > Mn_2_O_3_ > MnO_2_ > MnO. The higher-valence manganese oxides were shown to have stronger oxidation ability, larger sulfur capacity, and the advantage of producing elemental sulfur with high utilization value during the reaction. After selecting Mn oxides as the optimal H_2_S removal agents, an equilibrium component analysis of the regeneration process of the sulfided MnS was carried out. The results indicate that an oxygen amount that is 1.5 times that of MnS is the optimal dosage, and such an amount can oxidize all of the MnS at a relatively low temperature. Conversely, a diluted oxygen concentration can further reduce the temperature of the regeneration process, preventing the sintering of the regenerated desulfurizer and thus maintaining its reusability. This research provides a sufficient theoretical basis for the use of Mn oxides as active components of desulfurizers to remove H_2_S from blast furnace gas and for the regeneration of MnS after desulfurization.

## 1. Introduction

As a foundational pillar of the global economy, the steel industry contributed 1.89 billion tons to global crude steel production in 2023 [1,2,3]. The production process generates 1700~2500 m^3^ of blast furnace gas (BFG) per ton of steel [4], which translates to a substantial volume of byproduct gas requiring safe and efficient utilization. BFG, a byproduct of steel manufacturing processes, contains hydrogen, methane, and carbon monoxide [5], endowing it with significant energy utilization potential and establishing it as the primary fuel source for industrial furnaces, such as hot blast stoves and reheating furnaces [6,7,8].

However, H_2_S and other impurities not only corrode transmission pipelines, but also generate polluting gases (e.g., SO_2_) during combustion [9,10]. These contaminants severely endanger human health, environmental integrity, and industrial infrastructure, thereby impeding the efficient utilization of gaseous resources [11]. Therefore, the concentration of H_2_S should be limited at very low levels on various occasions [12,13]. For example, the acceptable environmental thresholds for H_2_S have been stipulated as 0.02–0.1 ppm [14]. To comply with increasingly rigorous emission regulations (i.e., EU BREF), advanced BFG treatment becomes critical for achieving energy conservation and emission reduction objectives [15,16].

Current H_2_S deep removal strategies primarily comprise adsorption [17], absorption [18], catalytic oxidation [19], and biological treatment [20]. Among these, adsorption desulfurization has emerged as the most promising technology due to its high-efficiency deep H_2_S removal capacity [21], encompassing activated carbon adsorption [22,23], microcrystalline material adsorption [24], and metal oxide adsorption [25,26,27]. Compared with activated carbon adsorption that requires continuous oxygen supplementation [28] and microcrystalline methods suffering from high implementation costs [29], metal oxide adsorption offers advantages, such as high sulfur removal efficiency and excellent selectivity [30]. Notably, the elemental sulfur generated during the process can be recycled for industrial applications, thereby promoting circular economy practices and being recognized by the international academic community as a highly viable technical pathway [31]. The development of high-efficiency desulfurizers and optimization of regeneration protocols are of profound significance for enhancing BFG utilization efficiency and advancing energy-saving and emission-reduction initiatives in the global steel industry [32,33].

## 2. H_2_S Removal and Regeneration Process by Metal Oxides

**Figure 1:**
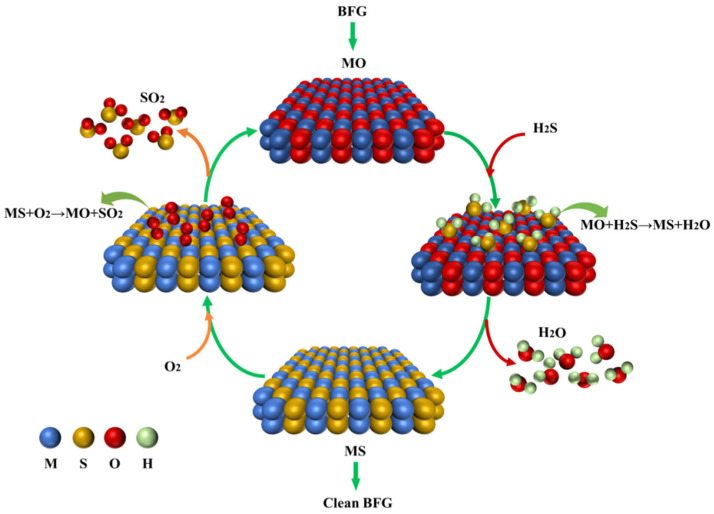
Flow chart of the H_2_S removal and regeneration process by metal oxides (M represents metal elements).

The H_2_S desulfurization and metal oxide regeneration process is schematically depicted in Figure 1. Initially, the metal oxide undergoes a reaction with the H_2_S present in BFG, yielding sulfide and water. Subsequently, oxygen introduction facilitates the oxidative regeneration of sulfide back to metal oxide, with SO_2_ concurrently produced as a byproduct.

Extensive investigations have been conducted on Fe-, Cu-, Ca-, and Zn-based, as well as composite, metal oxides as desulfurization sorbents for H_2_S abatement. Zhan et al. [30] reported α-Fe_2_O_3_ synthesis via MIL-101 (Fe) calcination at 500 °C, achieving H_2_S oxidation to sulfur and sulfate. However, this iron-based sorbent suffers from sintering issues under high-temperature conditions. In contrast, Wu et al. [34] introduced a microwave sulfidation protocol using activated carbon-supported Fe_2_O_3_, demonstrating optimal performance at 600 °C. Rezaei et al. [35] fabricated titanium silicate-supported CuO via ion exchange, which exhibited enhanced porosity and achieved sub-0.5 ppm desulfurization precision. Li et al. [8] developed a Cu-K-Co/AC catalyst through impregnation for simultaneous COS and H_2_S removal. Their results showed sulfur capacities of 90.59 mg/g for COS and 127.62 mg/g for H_2_S under 60 °C and 0.1 vol% O_2_. Mechanistic studies revealed that KOH-provided basic sites facilitated COS hydrolysis to H_2_S, while CuO/Cu_2_O lattice oxygen promoted H_2_S oxidation to elemental sulfur. Notwithstanding, sulfate accumulation was identified as the primary deactivation mechanism. Oh et al. [36] immobilized ZnO nanostructures (ZnO-nR vs. ZnO-nS) on cordierite–mullite supports via seed growth, demonstrating ZnO-nS superiority with 48.7 mg/g sulfur capacity and 75.4 min breakthrough time at 400 °C. This performance was attributed to improved surface coverage and crystallinity. Significantly, ZnO-nS retained 95% of its initial capacity after five regeneration cycles, indicating favorable mass transfer properties. Feng et al. [37] compared ZnO synthesis methods (room temperature solid-phase method vs. homogeneous precipitation method), revealing that the latter produced sorbents with superior textural properties (40.81 m^2^/g surface area vs. 33.20 m^2^/g) and regeneration efficiency. However, Zn volatility remained a critical limitation. Conversely, CaO-based sorbents [38] have been shown to exhibit thermal stability up to 1200 °C with reversible O_2_ regeneration capability. Li et al. [39] optimized MnₓOᵧ/Al_2_O_3_ via calcination parameters, identifying 900 °C/6 h treatment as optimal for maintaining 85 mg/g sulfur capacity over five cycles.

Composite metal oxides have emerged as promising alternatives. Cimino et al. [40] reported Cu_0.5_Zn_0.5_/Al_5_ composite oxides with 26.2 mg/g sulfur capacity (9× improvement over unmodified γ-Al_2_O_3_). Min et al. [25] demonstrated that Fe-Cu synergy in Fe-Cu/SBA-15 composites enhanced H_2_S adsorption through basic environment formation, achieving 74.08 mg/g capacity. Sánchez-Hervás et al. [41] developed ZnO-NiO/rGO composites, achieving complete 9000 ppmv H_2_S removal at 400 °C under industrially relevant conditions. The rGO matrix improved metal oxide dispersion (99.35 m^2^/g) and sulfidation selectivity. Kim et al. [42] synthesized coral-like Mn_2_O_3_/Fe_2_O_3_ nanocomposites, demonstrating 11.97 mg/g room-temperature capacity—4.8× higher than α-Fe_2_O_3_.

Notwithstanding these advancements, existing literature lacks systematic thermodynamic screening of metal oxides and precise regeneration product distribution control. This study addressed these gaps by implementing computational thermodynamic modeling to systematically evaluate periodic table metal oxides and to provide theoretical guidance for optimizing regeneration product distributions.

## 3. Thermodynamic Analysis

Notably, the selection of metal oxides as active components in H_2_S adsorbents is of paramount importance. In previous research [43], a systematic screening of metal oxides as active components for SO_2_ removal from flue gas was conducted via thermodynamic methods. Manganese oxides have been identified to exhibit high desulfurization activity across a broad temperature range, and their desulfurization performance remains unaffected by other gas components in flue gas. Notably, the MnSO_4_ generated during desulfurization can be regenerated into manganese oxides using H_2_, enabling cyclic utilization of the desulfurizer and recovering substantial sulfur crystals. Subsequent experiments [44] have confirmed that manganese-based metal oxides exhibit superior efficiency and sulfur capacity for flue gas desulfurization compared to other sorbents, with the desulfurization efficiency of the sorbent being significantly enhanced and the operational cost of desulfurizers for enterprises reduced accordingly.

Ideal candidates must exhibit high sulfur-fixation capacity, rapid reaction, robust mechanical stability, negligible side-reactions, and cost-effectiveness [45,46,47]. Therefore, prior to the experimental validation of H_2_S removal from blast furnace gas using metal-oxide-based desulfurizers, thermodynamic analysis was conducted to evaluate the potential candidates. Gibbs free energy serves as a critical parameter for determining reaction spontaneity and feasibility. For a generic reaction, the Gibbs free energy change (Δ*G*) was calculated via Equation (1):

(1) ΔG=−RTlnK+RTlnQ

The spontaneity of chemical reactions is governed by the Gibbs free energy change: Δ*G* < 0 denotes spontaneous forward progression, Δ*G* > 0 signifies non-spontaneity, and Δ*G* = 0 corresponds to equilibrium states [48]. Notably, temperature emerges as a critical variable in thermodynamic evaluations, and it is capable of reversing reaction spontaneity through thermal modulation.

HSC Chemistry (developed by Outotec, Finland) is a preeminent thermodynamic and chemical equilibrium simulation platform in process engineering and materials science. It is integrated with a thermochemical database encompassing over 28,000 compounds and advanced computational algorithms, which enables the prediction of reaction feasibility, phase equilibria, and energy requirements under user-defined conditions [49,50]. Key functionalities encompass the following: (1) calculation of Gibbs free energy (Δ*G*), enthalpy (Δ*H*), entropy (Δ*S*), and heat capacity (*Cp*) for chemical reactions; (2) equilibrium analysis for gaseous, liquid, solid, and aqueous systems; and (3) process simulation, involving optimization of temperature, pressure, and reactant stoichiometry. Notably, software was utilized to conduct comprehensive thermodynamic investigations on metal oxides. Westmoreland and Harrison [51] elucidated the thermodynamic behavior of H_2_S adsorption on the oxides of Cu, Zn, Fe, V, Mn, Ca, Mo, W, and Co. Abdalla et al. [52] investigated copper-based oxygen carriers for chemical looping air separation (CLAS) oxygen production. HSC software (v6.0) was employed to perform thermodynamic evaluations, identifying optimal conditions for oxygen generation and carrier oxidation while simulating oxygen equilibrium partial pressure. Lopez Ortiz et al. [53] utilized HSC software for thermodynamic modeling to determine the optimal reaction temperature between cobalt tungstate and methane. Jerndal et al. [54] utilized HSC software to perform thermal analysis, modeling the reactions of multiple oxygen carriers in chemical looping combustion. Their results demonstrated complete fuel gas conversion over copper, manganese, and iron oxides. Xuan et al. [43] employed HSC software for the thermodynamic modeling of a flue gas desulfurization (FGD) system, identifying manganese-based oxides as the optimal active components for chemical looping desulfurization. These materials exhibit distinct advantages, including environmental benignity, sintering resistance, and cost-effectiveness, alongside high sulfur-fixation capacity; chemical inertness toward CO_2_, H_2_O, and CO; and enhanced SO_2_ removal.

The Reaction Equation and Equilibrium Composition modules of HSC Chemistry 6.0 were employed to systematically investigate the thermodynamic behaviors of the H_2_S removal and desulfurizer regeneration processes. Using the Reaction Equation module, the Gibbs free energy changes in reactions at varying temperatures were calculated to evaluate the thermodynamic feasibility of H_2_S removal from blast furnace gas by various metal oxide desulfurizers. The Equilibrium Composition module was utilized to calculate reaction products under diverse operating conditions for both desulfurization and regeneration processes, thereby elucidating the dominant reaction mechanisms and optimizing process parameters [55]. This study systematically evaluated the reactivity of metal oxides with blast furnace gas components, leveraging thermodynamic principles. Notably, blast furnace gas [56] contains not only major constituents (CO, CO_2_, N_2_, H_2_, O_2_, and CH_4_), but also trace sulfur compounds (H_2_S and COS), which are quantified in Table 1. A total of 22 candidate metal oxides were selected from the periodic table, excluding radioactive, low-melting, or prohibitively expensive metals. The final set included Al, Ti, V, Cr, Mn, Fe, Co, Ni, Cu, Zn, Zr, Sb, Nb, Mo, Ta, W, Hf, Mg, Ca, Sr, Ba, and Cd.

## 4. Results and Discussion

The selected temperature range of 100–1000 °C encompasses the operational spectrum of blast furnace gas from generation to utilization, comprising the medium–low temperature fraction of blast furnace gas post-waste heat recovery and the high-temperature fraction of unpurified, uncooled hot coal gas [57,58,59,60,61,62]. The temperature range for H_2_S removal by metal oxides was established as follows: Desulfurization temperatures for H_2_S removal from BFG using metal oxide sorbents are contingent upon the specific sorbent chemistry, typically spanning a broad thermal regime. The temperatures at which metal oxide desulfurizers remove H_2_S from BFG vary with the type of desulfurizer, typically spanning a broad temperature interval. For instance, iron-based sorbents demonstrate optimal desulfurization efficiency at 100–200 °C, whereas zinc- and manganese-based materials are preferentially deployed within the 200–600 °C range [58,59]. Garces et al. [60] reported that commercial ZnO exhibits a direct correlation between sulfidation temperature (60–400 °C) and sorbent breakthrough time. Li et al. [61] experimentally evaluated Zn-Mn oxides supported on γ-Al_2_O_3_ as sorbents for H_2_S removal within the 350–600 °C range. The results demonstrated that 10 wt% Zn-Mn/γ-Al_2_O_3_ composites achieve a notable H_2_S adsorption capacity of 52 mg S/g-sorbent at 600 °C. At ultra-high temperatures (600–1000 °C), specialized treatment protocols are requisite to preserve sorbent stability [62]. The thermal tolerance of the desulfurization systems was established as follows: Fixed-bed reactors employing metal oxide sorbents are engineered to withstand operational temperatures ≤ 1000 °C [63]. Collectively, the 100–1000 °C temperature envelope encompasses the entirety of BFG processing stages, the optimal performance windows of metal oxide sorbents, and the thermal limits of infrastructure, thereby establishing a comprehensive operational framework.

### 4.1. Fixing-Sulfur Potentiality

For the fixing-sulfur potentiality of the metal oxides, the Fe, Ni, Cu, Zn, Sb, Mo, W, Ca, Sr, Ba, Cd, V, Mn, and Co oxides must have excellent characteristics of active components in the desulfurizer, which is consistent with previous studies. Manganese oxides (MnO_X_) have received special emphasis as an absorbent for element sulfur recovery. Fang et al. [64] investigated the H_2_S removal performance of activated carbon (AC) supported with various metal oxides. Their study revealed that the H_2_S removal efficiencies of the as-prepared catalysts followed a descending order: Mn/AC > Cu/AC > Fe/AC > Ce/AC > Co/AC > V/AC. Copper oxide (CuO) adsorbent has been extensively investigated as a promising candidate owing to its favorable thermodynamic properties and high H_2_S removal capacity. Wang et al. [65] evaluated the performance of three-dimensionally ordered macroporous (3DOM) CuO adsorbents for H_2_S removal at ambient temperature. The results demonstrated that the CuO adsorbents achieved a desulfurization efficiency exceeding 99% and a breakthrough sulfur capacity of 137 mg/g-adsorbent. Iron oxides derived from mining residues are regarded as promising adsorbents for H_2_S removal. Cristiano et al. [66] investigated the adsorption performance of nanostructured iron oxide (NIO) toward H_2_S at room temperature, whereby 100% removal efficiency was achieved and sustained for 5.6 h. Magnesium oxide (MgO) and zinc oxide (ZnO) sorbents are also reported in many flue gas desulfurization systems, which has prompted researchers to view them as promising adsorbents. Yang et al. [27] investigated the H_2_S removal capacities of MgO, ZnO, and composite MgₓZn1-X/AC adsorbents at ambient temperature. The results demonstrated that the sulfur capacities of monometallic MgO and ZnO were both inferior to those of the composite counterparts. Table 2 summarizes the desulfurization performance and cost [67] of common metal oxides for flue gas desulfurization. Evidently, the Mn oxides exhibited the lowest cost but highest sulfur capacity.

Metal oxides react with H_2_S via two distinct pathways, as shown in Equations (2) and (3) [68]:

(2) $$\mathrm{MO}+H_{2}S\to \mathrm{MS}+H_{2}O,$$

(3) $$\mathrm{MO}+H_{2}S\to \mathrm{MS}+H_{2}O+S$$

where M denotes a metallic element.

**Figure 2:**
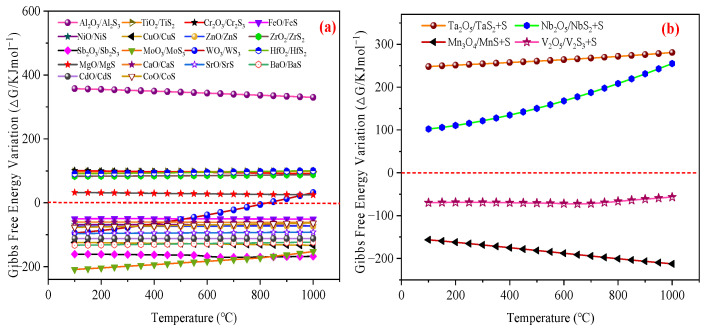
The fixing-sulfur potentiality of the 22 types of metal oxides when subjected to temperatures from 100 °C to 1000 °C: (**a**) The reaction of Equation (2). (**b**) The reaction of Equation (3).

The sulfur-containing reaction products include sulfides (MS) and elemental sulfur (S). Gibbs free energy calculations were performed to evaluate the sulfur-fixation capacity of the candidate metal oxides. Figure 2 illustrates the Δ*G* profiles for H_2_S reactions with 22 metal oxides. Notably, Figure 2a reveals that the Al, Ti, Cr, Zr, Hf, and Mg oxides exhibited positive Δ*G* values across the 100~1000 °C range, indicating thermodynamic infeasibility for H_2_S removal. Conversely, the Fe, Ni, Cu, Zn, Sb, Mo, Ca, Sr, Ba, Cd, and Co oxides demonstrated negative Δ*G* values, signifying spontaneous reactivity with H_2_S under these conditions. WO_3_ displayed temperature-dependent behavior, with spontaneous reactions occurring between 100~800 °C. The results presented in Figure 2b indicate that the Nb and Ta oxides exhibited positive Δ*G* values for the H_2_S reactions across the 100~1000 °C range, whereas the V and Mn oxides demonstrated negative Δ*G* values, signifying spontaneous reactivity under these conditions.

Collectively, the Al, Ti, Cr, Zr, Hf, Mg, Nb, and Ta oxides were excluded from active component selection due to their lack of reactivity with H_2_S. In contrast, the Fe, Ni, Cu, Zn, Sb, Mo, W, Ca, Sr, Ba, Cd, V, Mn, and Co oxides emerged as promising candidates for H_2_S removal applications.

### 4.2. Reacting with CO and CO_2_

**Figure 3:**
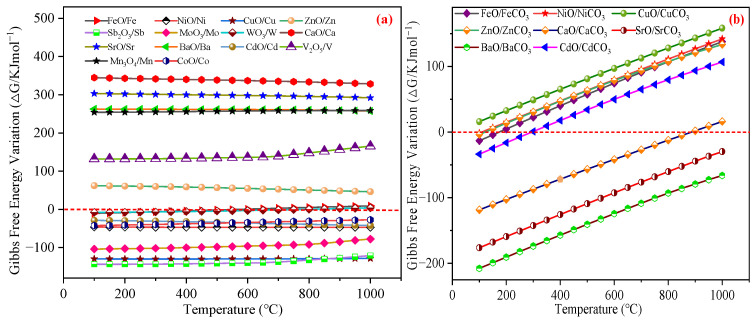
The ∆*G* variation of the 14 types of metal oxides under different temperatures: (**a**) The chemical reaction of the metal oxides with CO. (**b**) The chemical reaction of the metal oxides with CO_2_.

During the desulfurization processes, metal oxides may undergo reduction reactions with CO, generating trace metallic species that reduce oxide loading and impair H_2_S removal reactivity [69,70]. Thermodynamic spontaneity dictates that reactions proceed in the forward direction when Δ*G* < 0. Figure 3a reveals that the Fe, Ni, Cu, Sb, Mo, W, Cd, and Co oxides exhibited spontaneous CO interactions within the 100~1000 °C range, forming dissociative metallic phases that compromised the desulfurizer performance. Conversely, the Zn, Ca, Sr, Ba, V, and Mn oxides maintained positive Δ*G* values for CO reactions, indicating chemical inertness toward CO. These findings justified the exclusion of Fe, Ni, Cu, Sb, Mo, W, Cd, and Co oxides from active component selection.

CO_2_ ranked as the third most abundant constituent in the blast furnace gas, necessitating evaluation of its interaction with the metal oxides. During desulfurization, metal oxides may adsorb CO_2_ in addition to H_2_S, forming carbonates that induce irreversible deactivation of active sites [71,72,73].

Figure 3b illustrates the Δ*G* profiles for CO_2_ reactions with eight metal oxides. The Sb, Mo, W, V, Mn, and Co oxides were excluded from the analysis due to their chemical inertness toward CO_2_. Notably, the Sr and Ba oxides exhibited negative Δ*G* values across the 100~1000 °C range, while CaO and CdO demonstrated temperature-specific reactivity (100 °C~900 °C and 100~300 °C, respectively). These spontaneous carbonate-forming reactions compromised the H_2_S removal efficiency, leading to their elimination from active component selection. Conversely, CuO maintained positive Δ*G* values, indicating thermodynamic stability against CO_2_. For the Fe, Ni, Zn, and Cd oxides, Δ*G* increased with temperature, shifting the following reaction spontaneity thresholds: NiO and ZnO showed initial negative Δ*G* near 100 °C, FeO between 100~150 °C, and CdO between 100~250 °C before becoming non-spontaneous at elevated temperatures.

Collectively, the V, Mn, and Zn oxides emerged as optimal candidates as they resisted both the CO-induced reduction and CO_2_-driven carbonate formation, thereby maintaining structural integrity during the H_2_S removal.

### 4.3. Reacting with H_2_O

**Figure 4:**
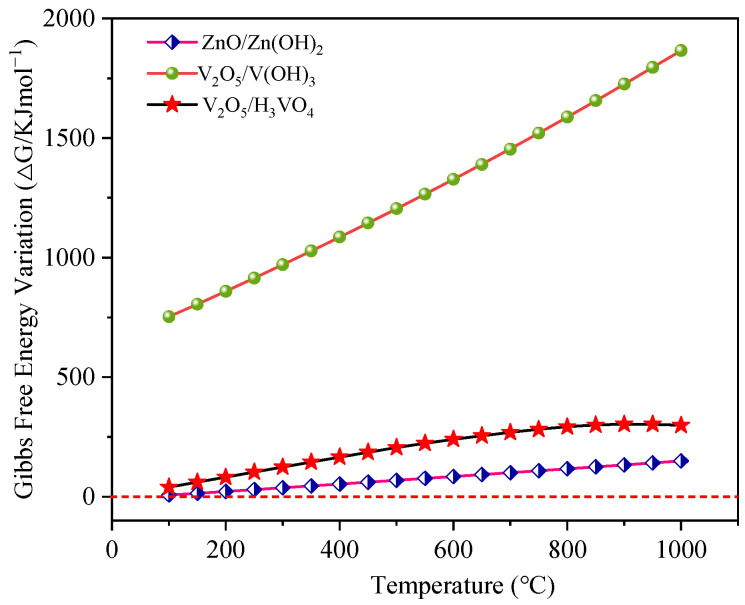
The changes in the Δ*G* of the Zn and V oxides at different temperatures (when reacting with H_2_O).

Blast furnace gas (BFG) contains significant amounts of saturated water, and the continuous cooling of the gas network leads to the formation of liquid water, which impacts the desulfurization process. Notably, metal oxides may react with H_2_O in BFG to form hydroxides. As Kariya et al. illustrated, the chemical heat storage systems based on CaO/H_2_O were expected to utilize waste heat for the purpose of heat storage [74]. Sol-gel based on MgO/H_2_O, which is known to be a clean, environmentally friendly, inexpensive and readily prepared process, has been used to improve the corrosion resistance of anodized magnesium alloys [75]. Figure 4 illustrates the Gibbs free energy changes for the reaction of each metal oxide with H_2_O. Due to the high chemical stability of Mn_3_O_4_, this oxide exhibits no appreciable reaction with H_2_O, yielding neither hydroxides nor acids; consequently, Mn_3_O_4_ is absent from Figure 4. Analysis of Figure 4 reveals that the Δ*G* for the reaction of ZnO with H_2_O remained positive across 100~1000 °C, indicating that the spontaneous formation of Zn(OH)_2_ did not occur. Conversely, for V_2_O_5_, the Δ*G* values for both reactions—formation of vanadium hydroxide (V(OH)_3_) and vanadic acid (H_3_VO_4_)—remained positive within the same temperature range, demonstrating that V_2_O_5_ does not interact with water under these conditions. Therefore, it can be concluded that H_2_O in BFG has negligible influence on the removal of H_2_S by V-, Mn-, and Zn-based oxides, and thus the active components were not excluded from consideration.

### 4.4. Reacting with CH_4_

**Figure 5:**
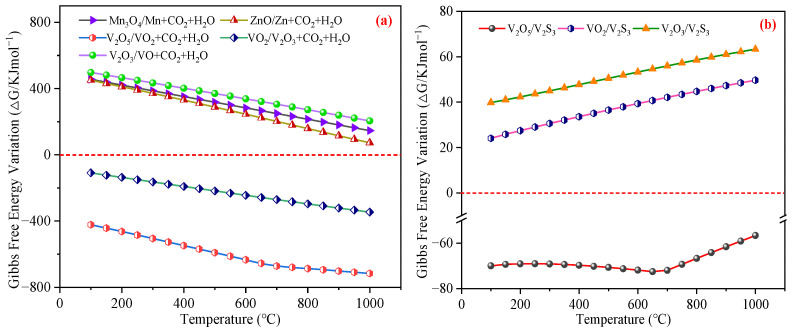
(**a**) The changes in the Δ*G* of the Zn, Mn, and V oxides at different temperatures (when reacting with CH_4_). (**b**) The changes in the Δ*G* of the V_2_O_5_, VO_2_, and V_2_O_3_ at different temperatures (when reacting with H_2_S).

Blast furnace gas contains trace amounts of CH_4_, necessitating evaluation of its potential impact on metal oxides. CH_4_ may initiate reduction reactions with V, Mn, and Zn oxides, potentially compromising desulfurization performance [76]. Alalwan et al. [77] focused on the CH_4_ activation reaction on the surface of metal oxide nanoparticles. Through in situ diffuse reflectance infrared Fourier transform spectroscopy (DRIFTS) and other characterization methods, the reaction pathways and mechanisms of CH_4_ on the surfaces of CoO, CuO, and α-Fe_2_O_3_ are revealed, providing key insights for processes such as chemical looping combustion (CLC) and methane carbon dioxide reforming. Figure 5a demonstrates that Mn_3_O_4_ and ZnO maintained positive Δ*G* values for the CH_4_ interactions across 100~1000 °C, indicating thermodynamic stability. Conversely, V_2_O_5_ undergoes sequential reduction to VO_2_ and V_2_O_3_, though the final step to VO remains non-spontaneous. These findings confirm VO_2_ and V_2_O_3_ as the primary reduction products. Notably, Figure 5b reveals that only V_2_O_5_ exhibits spontaneous H_2_S reactivity within the 100~1000 °C range, while its reduction products (VO_2_ and V_2_O_3_) remain inert. This highlights the deleterious effect of CH_4_-induced V_2_O_5_ reduction, which not only generates inactive phases, but also produces CO_2_ as a byproduct. Given the irreversible deactivation of V_2_O_5_ by CH_4_, it is excluded from active component selection. In contrast, Mn and Zn oxides demonstrate chemical inertness toward CH_4_, establishing their superiority over vanadium-based materials.

### 4.5. Comparison of the Zinc-Manganese-Based Oxides

**Figure 6:**
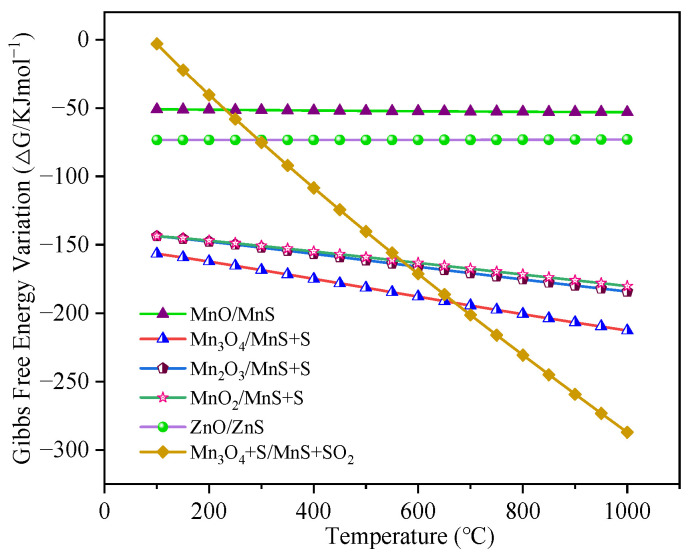
The Δ*G* changes in the desulfurization of the Zn and Mn oxides at different temperatures.

Following thermodynamic screening based on blast furnace gas composition, the Mn and Zn oxides were identified as optimal active components. Further analysis of their valence-state oxides revealed significant differences. While ZnO remained thermodynamically stable, other zinc oxides (e.g., ZnO_2_ and Zn_2_O) exhibited instability. Mn’s 3d^5^4s^2^ valence electron configuration endows its oxides with half-filled d orbitals across multiple oxidation states, thereby facilitating electron transfer and redox processes [78]. Conversely, Mn oxides demonstrate stronger oxidative capacity and diverse valency (MnO, Mn_3_O_4_, Mn_2_O_3_, and MnO_2_) compared to monovalent ZnO. Figure 6 illustrates the Δ*G* profiles for desulfurization reactions involving Zn and Mn oxides, encompassing the following pathways:

MnO + H_2_S(g) = MnS + H_2_O(g), (4)

Mn_3_O_4_ + 4H_2_S(g) = 3MnS + 4H_2_O(g) + S, (5)

Mn_2_O_3_ + 3H_2_S(g) = 2MnS + 3H_2_O(g) + S, (6)

MnO_2_ + 2H_2_S(g) = MnS + 2H_2_O(g) + S, (7)

ZnO + H_2_S(g) = ZnS + H_2_O(g), (8)

5S + Mn_3_O_4_ = 3MnS + 2SO_2_(g). (9)

Figure 6 reveals that all of the evaluated reactions Equations (4)–(8) exhibited negative ΔG values across 100~1000 °C, confirming spontaneous H_2_S removal by ZnO and the four Mn oxides. Notably, lower Δ*G* values indicate stronger sulfur-fixation capacity. These reactions are ranked by spontaneity: Mn_3_O_4_ > Mn_2_O_3_ > MnO_2_ > ZnO > MnO. Thermodynamic analysis indicates higher-oxidation-state Mn oxides demonstrate significantly greater H_2_S reactivity, with stronger oxidation capacity and higher sulfur-fixation potential, compared to ZnO and MnO [79].

**Figure 7:**
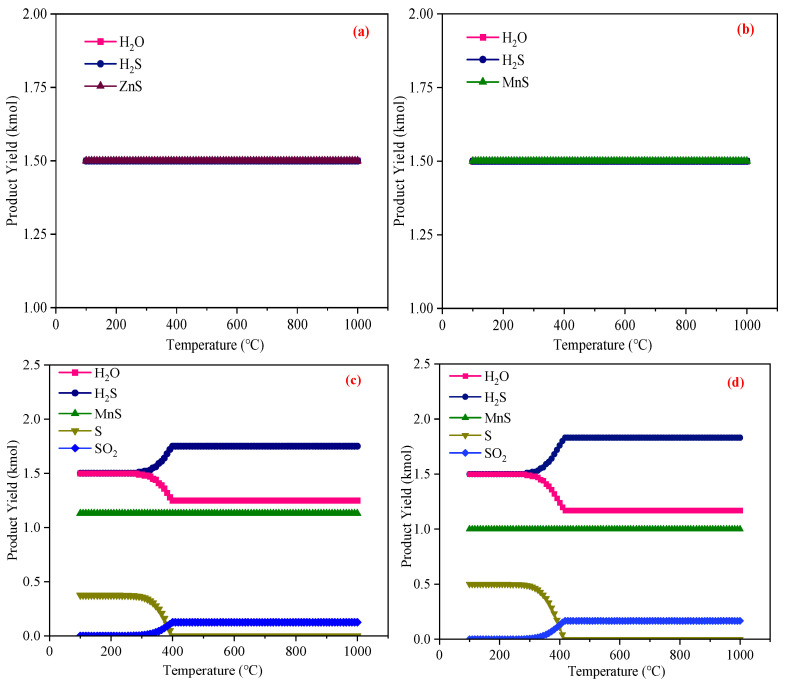

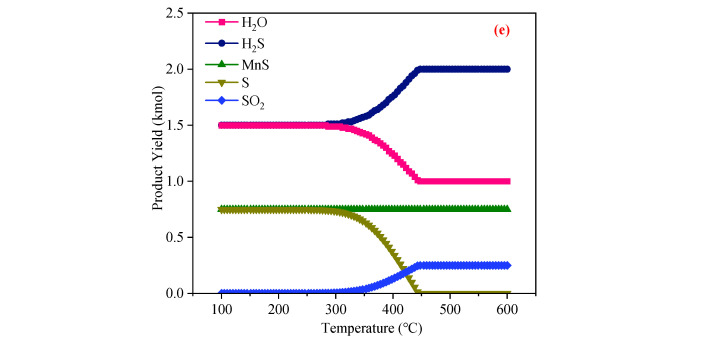
The production of various substances during the H_2_S removal process of Zn and Mn oxides: (**a**) 1.5 kmol of ZnO, (**b**) 1.5 kmol of MnO, (**c**) 0.375 kmol of Mn_3_O_4_, (**d**) 0.5 kmol of Mn_2_O_3_, and (**e**) 0.75 kmol of MnO_2_.

Figure 7 presents the results of an equilibrium component analysis for the reactions between ZnO, MnO, Mn_3_O_4_, Mn_2_O_3_, and MnO_2_ with H_2_S at temperatures ranging from 100~1000 °C. To mimic real-world desulfurization processes more accurately, an excess of H_2_S was employed, with the amount of H_2_S in the reactants set at 3 kmol (3% concentration). Based on Equations (4)–(8), the quantities of each oxide were determined to ensure that all oxides could remove an equal amount (1.5 kmol) of H_2_S under H_2_S-excess conditions: 1.5 kmol of ZnO, 1.5 kmol of MnO, 0.375 kmol of Mn_3_O_4_, 0.5 kmol of Mn_2_O_3_, and 0.75 kmol of MnO_2_. It was demonstrated that higher-valence Mn atoms can transfer more electrons, enabling them to remove more H_2_S and thus possess a larger sulfur capacity, with Mn_3_O_4_ exhibiting the greatest sulfur capacity among them.

Notably, the data from Figure 7a,b indicate that temperature had no significant impact on the reactions of ZnO and MnO with H_2_S, as ZnS, MnS, and H_2_O were consistently produced across the 100~1000 °C range. Conversely, as shown in Figure 7c, the sulfur-containing products of the reaction between Mn_3_O_4_ and H_2_S included MnS, S, and SO_2_. From 100~300 °C, reaction Equation (5) dominated, yielding 1.125 kmol of MnS, 0.375 kmol of S, and 1.5 kmol of H_2_O. As the temperature rose from 300 °C to 400 °C, the amount of S in the products decreased gradually, while that of SO_2_ increased, and the amount of H_2_S removed by Mn_3_O_4_ also declined. By examining the Δ*G* change in reaction Equation (9), as shown in Figure 6 (the yellow line in Figure 6), it can be elucidated that the S generated from the reaction between Mn_3_O_4_ and H_2_S could be further oxidized to SO_2_, where S and H_2_S are in a competitive relationship as reducing agents. With increasing temperature, the Δ*G* of Equation (9) drops significantly. Moreover, S volatilizes at higher temperatures, and its reactivity is greatly enhanced, making it more prone to oxidation to SO_2_. Therefore, above 400 °C, elemental S is absent from the products, and the consumption of Mn_3_O_4_ by S leads to a reduction in the amount of H_2_S removed by Mn_3_O_4_.

The product types and change trends shown in Figure 7d,e are generally similar to those in Figure 7c. In the low-temperature range (100~300 °C), reactions Equations (6) and (7) dominated, and both were able to generate an amount of elemental S equal to their own consumption. Therefore, when using high-valence Mn oxides for H_2_S removal, the temperature should not exceed 300 °C. At this temperature, elemental S can be stably produced, which can be reused as a chemical raw material, presenting significant economic value [80,81]. Additionally, high-valence Mn oxides remove a larger amount of H_2_S below 300 °C. It should be noted that MnO_2_ is unstable at high temperatures, undergoing thermal decomposition above 600 °C and being reducible by CO [82,83]. If MnO_2_ is employed for H_2_S removal from blast furnace gas, reaction conditions should be carefully controlled to minimize its thermal decomposition and reaction with CO.

In conclusion, high-valence Mn oxides possess advantages such as stronger oxidizing ability, larger sulfur capacity, and the production of elemental S with high re-utilization value. Therefore, the Mn oxides, with Mn_3_O_4_ exhibiting the highest sulfur-fixation potential and the largest sulfur capacity, were ultimately selected as the optimal desulfurizers.

The performance of different desulfurizers was compared, as shown in Table 3. Notably, the zinc-based sorbent exhibited high desulfurization efficiency but came with a high cost. Its desulfurization and regeneration processes require high temperature, and it exhibits inferior performance at low temperatures, thereby restricting its application in certain scenarios [26,36,60,84]. Conversely, iron oxide demonstrates low cost but moderate reactivity [85] as its performance is often constrained by poor structural stability and insufficient active sites [16,86,87,88].

Activated carbon relies on its high specific surface area and well-developed pore structure to achieve superior adsorption capacity [89]; however, its poor sulfur capacity and short breakthrough time remains suboptimal [27], necessitating metal impregnation or surface modification to enhance performance [9,90]. Critically, its regeneration process involves high temperatures and induces structural damage, thereby significantly increasing operational costs. By contrast, manganese oxide demonstrated the optimal balance of desulfurization efficiency, sulfur capacity, regenerative performance, and long-term economic viability [61,64,79], rendering it a suitable candidate for BFG desulfurization.

Furthermore, the Mn oxides outperformed other advanced materials like MOFs or perovskites in sulfur capture. Firstly, the H_2_S removal by the Mn oxides predominantly involved chemical adsorption and oxidation reactions, which are characterized by low activation energy [61]; consequently, the reaction kinetics were notably faster than those of MOFs (which rely on physical adsorption). Secondly, Mn oxides benefit from abundant raw materials (e.g., pyrolusite and battery waste) and straightforward preparation protocols [91], whereas MOFs and perovskites necessitate precious metals or intricate synthetic procedures (coupled with high regeneration energy demands) [92]. Thirdly, Mn oxides exhibit structural stability across 200–600 °C [71]; conversely, MOFs frameworks undergo collapse at > 300 °C. Notably, Mn oxides demonstrate robust tolerance toward common BFG impurities (e.g., CO and CO_2_), while perovskites, such as LaCoO_3_, are prone to sulfur poisoning and deactivation in sulfidic atmospheres. Thus, Mn oxides outweigh other materials in industrial sulfur capture applications owing to their rapid reaction kinetics, cost-effectiveness, and superior interference resistance.

In dry desulfurization systems, the surface area and porosity of desulfurizers also play a decisive role in determining H_2_S removal efficiency and adsorption capacity. Specifically, a larger specific surface area facilitates the provision of additional active sites, thereby accelerating chemical interactions between H_2_S and metal oxides; additionally, highly porous architectures promote gas diffusion into desulfurizer interiors, enhancing sulfur capacity and extending breakthrough duration. Thus, microstructural tuning via the design of novel desulfurizers represents a crucial strategy for performance enhancement [1,37].

## 5. Control of Reaction Products in the Regeneration Process of Desulfurizers

Manganese-based desulfurizers generate MnS during H_2_S removal, which can be regenerated via oxidation with O_2_. To evaluate the potential chemical reactions between MnS and O_2_ in the regenerator, HSC Chemistry 6.0 was employed to determine the reaction feasibility and to perform equilibrium composition analysis. Specifically, the reactions and equilibrium products were analyzed across a temperature range of 200~1200 °C at standard atmospheric pressure.

Calculations of the Gibbs free energy changes using HSC Chemistry 6.0 revealed the following primary reactions during MnS regeneration:

MnS + 2O_2_(g) = MnSO_4_, (10)

MnS + 1.5O_2_(g) = MnO + SO_2_(g), (11)

2MnO + 2SO_2_(g) + O_2_(g) = 2MnSO_4_, (12)

MnS + 3MnSO_4_ = 4MnO + 4SO_2_(g), (13)

MnS + O_2_(g) = Mn + SO_2_(g), (14)

2Mn + O_2_(g) = 2MnO, (15)

2MnS + O_2_(g) = 2MnO + 2S, (16)

S + O_2_(g) = SO_2_(g), (17)

2SO_2_(g) + O_2_(g) = 2SO_3_(g), (18)

SO_3_(g) + MnO = MnSO_4_, (19)

2S = S_2_(g), (20)

6MnO + O_2_(g) = 2Mn_3_O_4_, (21)

4MnO + O_2_(g) = 2Mn_2_O_3_, (22)

2MnO + O_2_(g) = 2MnO_2_, (23)

4Mn_3_O_4_ + O_2_(g) = 6Mn_2_O_3_, (24)

Mn_3_O_4_ + O_2_(g) = 3MnO_2_, (25)

2Mn_2_O_3_ + O_2_(g) = 4MnO_2_, (26)

3MnS + 5O_2_(g) = Mn_3_O_4_ + 3SO_2_(g), (27)

2MnS + 3.5O_2_(g) = Mn_2_O_3_ + 2SO_2_(g), (28)

MnS + 2O_2_(g) = MnO_2_ + SO_2_(g), (29)

Mn_3_O_4_ + 3SO_2_(g) + O_2_(g) = 3MnSO_4_, (30)

2Mn_2_O_3_ + 4SO_2_(g) + O_2_(g) = 4MnSO_4_, (31)

MnO_2_ + SO_2_(g) = MnSO_4_, (32)

MnS + 5MnSO_4_ = 2Mn_3_O_4_ + 6SO_2_(g), (33)

MnS + 7MnSO_4_ = 4Mn_2_O_3_ + 8SO_2_(g). (34)

**Figure 8:**
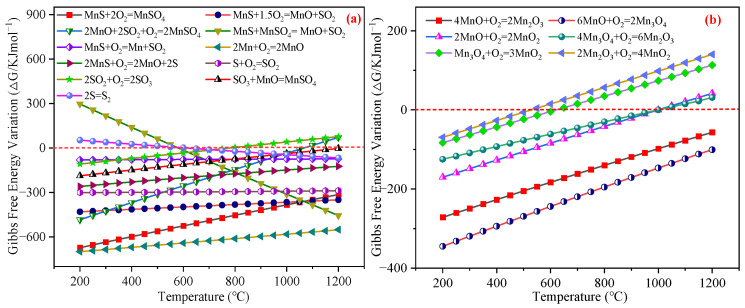
The changes in the Δ*G* of the various reactions during the following MnS regeneration processes: (**a**) involving MnO only, and (**b**) when MnO continued to be oxidized into high valence oxides.

Reactions Equations (21)–(26) involved the further oxidation of MnO to higher-oxidation-state oxides, which gave rise to the subsequent Reactions Equations (27)–(34). When considering MnO alone, Figure 8a illustrates the Δs*G* profiles for the MnS regeneration reactions. Figure 8b presents the Δ*G* changes for the MnO oxidation pathways. Thermodynamic analysis confirmed the thermal instability of MnO_2_ as Reactions Equations (25) and (26) exhibited a sign change in Δ*G* near 600 °C, indicating MnO_2_ decomposition at this temperature threshold.

**Figure 9:**
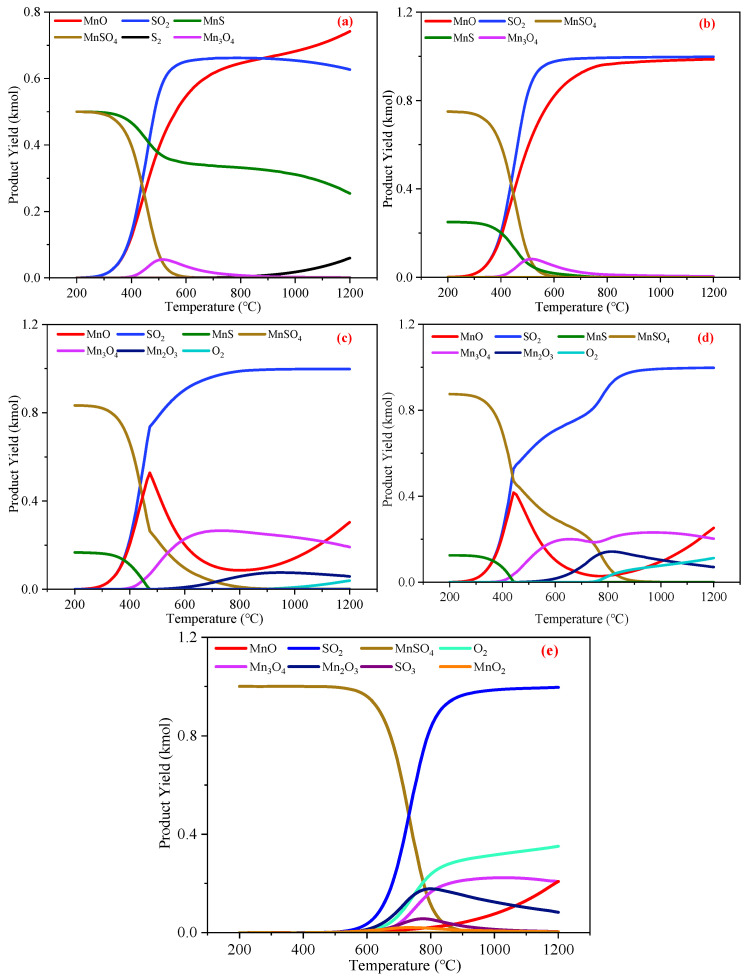
The production of various substances during the regeneration process of 1 kmol of MnS: (**a**) 1 kmol concentration of 5% oxygen, (**b**) 1.5 kmol concentration of 5% oxygen, (**c**) 1.67 kmol concentration of 5% oxygen, (**d**) 1.75 kmol concentration of 5% oxygen, and (**e**) 2 kmol concentration of 5% oxygen.

The regeneration of MnS is influenced by temperature variations, O_2_ stoichiometry, and concentration. Reaction Equation (11) specified that the complete oxidation of 1 kmol MnS to MnO and SO_2_ necessitates 1.5 kmol O_2_. Using MnO formation as a model, reactions during regeneration were analyzed, as shown in Figure 9a,b, revealing that the products were governed by reactions Equations (10)–(13).

Notably, Equations (10) and (11) exhibited robust negative Δ*G* across 200~1200 °C with minimal temperature dependence, whereas Equations (12) and (13) demonstrated pronounced temperature sensitivity. Equation (13)’s Δ*G* decreased sharply with temperature elevation, transitioning to negative near 600 °C, which indicates MnSO_4_ undergoes spontaneous decomposition with MnS to form MnO and SO_2_ at ≥600 °C.

Equation (12) maintained negative Δ*G* between 200~1000 °C but increased with temperature, reflecting stronger reactivity at lower temperatures. Since Equations (11) and (12) represented sub-reactions of Equation (10), the MnO and SO_2_ generated by Equation (11) at 200 °C further reacted via Equation (12) to form MnSO_4_. Consequently, Equation (10) dominated at low temperatures, yielding 0.75 kmol of MnSO_4_ and 0.25 kmol of unreacted MnS. As the temperature increased, Equation (12)’s Δ*G* elevation reduced MnSO_4_ and unreacted MnS while increasing MnO and SO_2_. When the temperature reached 600 °C, MnSO_4_ reacted with MnS to decompose into MnO and SO_2_. It can be seen that Equations (10) and (13) are components of Equation (11); as such, at high temperatures, the reaction was mainly dominated by Equation (11), and the products were MnO and SO_2_.

During MnS oxidation regeneration, reactions Equations (14), (16), and (18) generated metallic Mn, elemental S, and SO_3_ gas, respectively. These products were rapidly converted: Mn and S oxidized to MnO and SO_2_, while SO_3_ reacted with MnO to form MnSO_4_. Specifically, Equations (14)–(17) represented sub-reaction pathways for Equation (11), and Equations (18) and (19) served as sub-reactions for Equation (13). Given the thermodynamic favorability of Equations (15), (17) and (19), Mn, S, and SO_3_ acted as transient intermediates and, thus, do not appear in Figure 9b. Reaction Equation (20) was restricted to high-temperature, oxygen-deficient environments. Notably, S preferentially reacted with O_2_ over forming S_2_ gas, resulting in trace S_2_ gas formation at elevated temperatures only. Figure 9a illustrates partial S_2_ gas generation at high temperatures due to O_2_ limitation.

Under O_2_-sufficient conditions, MnO undergoes further oxidation. Reactions Equations (21)–(26) describe the progression of Mn oxides to higher oxidation states, initiating secondary reactions Equations (27)–(34). Figure 9b shows Mn_3_O_4_ formation above 400 °C, indicating partial MnO oxidation. The limited extent of MnO oxidation likely stems from the 1.5 kmol O_2_ dosage (matching Equation (11) stoichiometry for 1 kmol of MnS oxidation), which may be insufficient to drive complete oxidation to higher valence states.

The molar ratio of reactants specified in Equation (27) was adopted to elevate the O_2_ dosage to 1.67 kmol for component analysis simulation, as presented in Figure 9c. The observed phenomenon clearly demonstrates that the MnO content decreased rapidly with increasing temperature, while Mn_3_O_4_ and Mn_2_O_3_ concentrations increased. This trend elucidates that augmented O_2_ supply promotes further oxidation of MnO to higher-valence manganese oxides. Notably, MnO_2_ absence in products can be attributed to its thermal decomposition at elevated temperatures. The accelerated MnO consumption at higher temperatures is primarily ascribed to enhanced oxidation kinetics and oxygen reactivity, which are both temperature-dependent. Conversely, when temperature exceeds 900 °C, MnO reaccumulates with concomitant O_2_ evolution, presumably due to the thermodynamic inhibition of exothermic MnO oxidation under such conditions.

Following the stoichiometric ratios defined in Equations (10), (28), and (29), O_2_ dosages were further increased to 1.75 kmol and 2 kmol, yielding results shown in Figure 9d,e. Figure 9d exhibits similar trends to Figure 9c, but with systematically reduced MnO content and earlier O_2_ evolution. Significantly, Figure 9e demonstrates that, under O_2_-sufficient conditions, the Mn_3_O_4_ and Mn_2_O_3_ concentrations and their growth rates surpassed those of MnO, albeit they were accompanied by minor SO_3_ formation. This indicates that excess O_2_ not only facilitates MnO oxidation, but also promotes SO_2_ oxidation.

Although elevated O_2_ levels enhance MnO oxidation, the following critical consideration arise: increasing O_2_ dosage delays MnSO_4_ elimination until temperatures exceed 900 °C. Since MnSO_4_ is an undesirable byproduct in the regeneration process, such high-temperature conditions pose risks of manganese oxide sintering and equipment degradation. Conversely, insufficient O_2_ supply (e.g., 1 kmol of O_2_) leads to incomplete oxidation, as shown by the MnS presence in Figure 9a, which violates regeneration requirements.

Collectively, these results posit that 1.5 kmol of O_2_ per kmol of MnS represents the optimal dosage for regeneration. This configuration yields substantial MnO and SO_2_ production while avoiding excessive temperature elevation and associated sintering risks. Both O_2_ deficiency and excess introduce undesirable outcomes: incomplete oxidation versus operational challenges.

**Figure 10:**
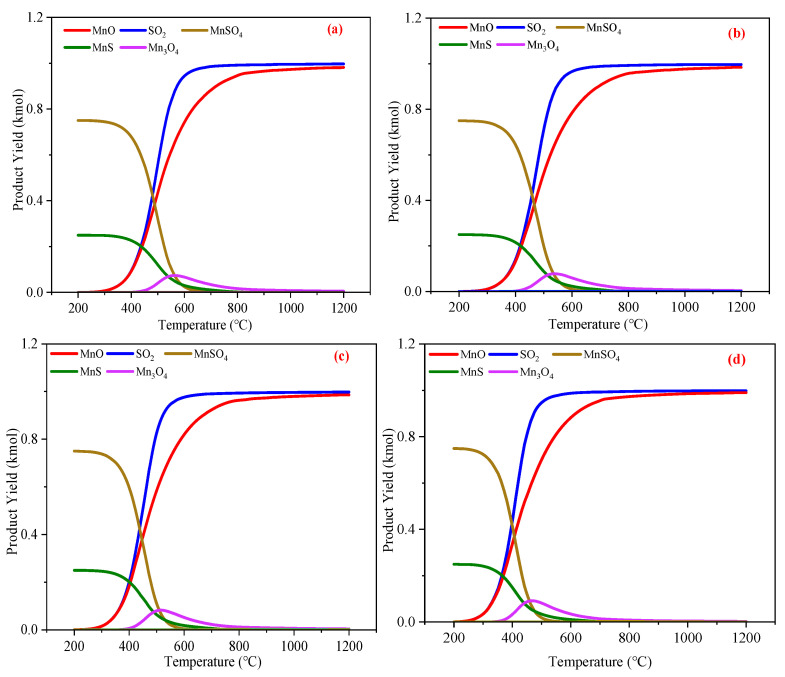
The production of various substances during the regeneration process of 1 kmol of MnS: (**a**) 1.5 kmol concentration of 20% oxygen, (**b**) 1.5 kmol concentration of 10% oxygen, (**c**) 1.5 kmol concentration of 5% oxygen, and (**d**) 1.5 kmol concentration of 1% oxygen.

The optimal oxygen dosage for MnS oxidative regeneration was determined to be 1.5 kmol. Subsequent equilibrium composition analysis of the regeneration process under varying oxygen concentrations, as depicted in Figure 10, reveals that the reaction products exhibited consistent species, and the curve variations were largely comparable. This observation demonstrates that lower oxygen concentrations slightly reduced the temperature thresholds for MnS and MnSO_4_ disappearance, which is consistent with previous studies [93,94,95]. The MnS-O_2_ reaction is highly exothermic with elevated reactivity; excessively high oxygen concentrations may trigger violent reactions leading to localized overheating, thereby increasing risks of desulfurizer sintering and equipment damage [96,97]. Therefore, oxygen dilution mitigates MnO sintering and extends operational lifespan.

Collectively, providing 1.5-fold stoichiometric oxygen achieves complete MnS oxidation at reduced temperatures. Oxygen concentration dilution further lowers regeneration temperatures while preventing thermal runaway-induced sintering and equipment degradation. At 1.5-fold stoichiometry with 5% O_2_, full MnS conversion was achieved at 700 °C, yielding MnO, SO_2_, and minor Mn_3_O_4_. This characteristic is highly compatible with the characteristics of industrial off-gas. For example, the flue gas of steel plants (usually containing 5~15% O_2_), or the low-oxygen gas in the tail gas of chemical plants, can be directly used as the regeneration gas source, avoiding the additional energy consumption of pure oxygen preparation or high-concentration oxygen dilution. Compared with the traditional method of using high-purity oxygen, the use of industrial waste gas as the regeneration gas source can significantly reduce the operating cost.

For industrial implementation, the SO_2_-rich tail gas can be directly channeled into a sulfuric acid production unit. The contact process typically achieves > 99% SO_2_ conversion efficiency, generating commercial-grade sulfuric acid that can be reused in steel pickling, water treatment, or sold as a byproduct to offset desulfurization costs. For example, each ton of SO_2_ processed yields approximately 1.5 tons of sulfuric acid, creating an economic benefit of ~USD 200–300 per ton of MnS regenerated, while simultaneously ensuring SO_2_ emissions comply with China’s Iron and Steel Industry Air Pollutant Emission Standards.

## 6. Conclusions

This study systematically employed thermodynamic analysis to screen suitable metallic desulfurizers and to investigate the reaction product regulation during desulfurizer regeneration. Key findings are presented as follows:

(1) Thermodynamic evaluations of reactions between blast furnace gas components and candidate metal oxides identified manganese oxides as the most promising H_2_S sorbent. These materials exhibit advantages, including non-radioactive characteristics; cost-effectiveness; high sulfur retention capacity; chemical inertness toward CO_2_, CO, and CH_4_; and unique stability across multiple high-valence Mn oxide phases.
(2) Comprehensive characterization of four manganese oxide phases revealed sulfur retention capacities in the following descending order: Mn_3_O_4_ > Mn_2_O_3_ > MnO_2_ > MnO. High-valence Mn oxides demonstrated superior oxidation potential, larger sulfur storage capacities, and the ability to generate elemental sulfur with high industrial utility. Notably, MnO_2_ underwent thermal decomposition at elevated temperatures and reacted with CO, necessitating stringent control of the reaction parameters to suppress undesirable side reactions.
(3) Compositional analysis of MnS oxidation indicated that 1.5 kmol of O_2_ per kmol of MnS represents the optimal stoichiometry for producing MnO and SO_2_. Oxygen deficiency resulted in incomplete regeneration, whereas excess oxygen induced significant temperature excursions, leading to manganese oxide sintering and accelerated equipment degradation. Oxygen concentration dilution effectively mitigated thermal runaway while reducing regeneration temperatures. Under 1.5-fold stoichiometric conditions with 5% O_2_, complete MnS conversion was achieved above 700 °C, yielding MnO, SO_2_, and trace amounts of Mn_3_O_4_.
(4) The present study primarily relied on thermodynamic equilibrium calculations, which do not account for kinetic factors (e.g., reaction rates and mass transfer limitations) that may influence practical H_2_S removal and regeneration processes. In our future work, experimental validation will be carried out to bridge the gap between thermodynamic predictions and practical applications, ensuring the reliability of Mn oxide-based desulfurizers in real blast furnace gas treatment systems.

## Figures and Tables

**Table 1:** Sulfur content of the blast furnace gas (mole fraction) unit: μmol/mol.

Component	Hydrogen Sulfide (H_2_S)	Carbon Based Sulfur (COS)	Methyl Mercaptan (CH_3_SH)	Thiophene (C_4_H_4_S)	Other
Content	30.7	73.8	0.0323	0.0176	<0.01

**Table 2:** The main studies on the desulfurization performance and cost of metal oxides.

Metal Oxide	Desulfurization Efficiency/%	Sulfur Capacity /(mg/g-Sorbent)	Experimental Temperature/°C	H_2_S Concentration /vol.%	Desulfurizer Cost /(Yuan/Kg-Sorbent)	Refs.
Manganese oxide	>99	142	180	0.3	12	[64]
Vanadium oxide	98	6.05	180	0.3	88	[65]
Copper oxide	>99	94–137	30–80	0.035	150	[65]
Iron oxide	100	1.0–2.5	25	0.02–0.05	790	[66]
Magnesium oxide	99	32.7	30	0.06	180	[27]
Zinc oxide	99	38.5	30	0.06	35	[27]

**Table 3:** Comparative analysis of the performance of the different desulfurizers.

Sorbents	Efficiency (%)	Capacity (mg/g)	Reusability	Comprehensive Cost	Refs.
Zinc-based	>99%	48.7	No	Difficult to regenerate, hazardous waste	[26,36,60,84]
Iron-based	>99%	16	No	High replacement	[16,85,86,87,88]
Activated carbon	~95%	3	No	Prone to blockage	[9,27,89,90]
Manganese-based	~99%	142	Yes	Long service life	[61,64,79]

## Data Availability

The original contributions presented in this study are included in the article. Further inquiries can be directed to the corresponding authors.

## References

[1] World Steel Association (2024). World Steel in Figures 2024. https://worldsteel.org/data/world-steel-in-figures-2024.

[2] Wang P.T., Xu Q.C., Wang F.Y., Xu M. (2025). Study on the coupling of the iron and steel industry with renewable energy for low-carbon production: A case study of matching steel plants with photovoltaic power plants in China. Energy.

[3] Yin R.Y., Shangguan F.Q., Cui Z.F. (2025). Research on low-carbon development strategies in steel industry: Review and prospect. Chin. Metall..

[4] Du X.W., Sun J.L., Yang W.M. (2023). Techno-economic simple analysis on desulfurization and its status of blast furnace gas. Guangdong Chem. Ind..

[5] Wei F.J., Zhang X.X., Liao J.J., Guo J.W., Bao W.R., Chang L.P. (2022). Desulfurization mechanism of an excellent Cu/ZnO sorbent for ultra-deep removal of thiophene in simulated coke oven gas. Chem. Eng. J..

[6] Cao R., Ning P., Wang X.Q., Wang L.L., Ma Y.X., Xie Y.B., Zhang H., Qu J.X. (2022). Low-temperature hydrolysis of carbonyl sulfide in blast furnace gas using Al_2_O_3_-based catalysts with high oxidation resistance. Fuel.

[7] Guo Y.H. (2020). Current station and tendency of purification and upgrading of blast furnace gas. J. Iron Steel Res..

[8] Li X., Wang X.Q., Wang L.L., Ning P., Ma Y.X., Zhong L., Wu Y., Yuan L. (2022). Efficient removal of carbonyl sulfur and hydrogen sulfide from blast furnace gas by one-step catalytic process with modified activated carbon. Appl. Surf. Sci..

[9] Zhao Y.Q., Dou J.X., Li H., Dai R.J., Bai H.C., Rish S.K., Chen X.X., Xiao X.X., Yu J.L. (2022). Low-cost Na2S-EG-MTPB deep eutectic solvents absorb SO_2_ effectively at a high temperature in flue gas. Sep. Purif. Technol..

[10] Jiang L.L., Zhao Y.S., Meng Y.M., Tu S.H., Chen Z.Y., Yu H.T., Hou X.G. (2021). Numerical simulation of co-injection of pulverized coal and blast furnace gas separated by a membrane. Ironmak. Steelmak..

[11] Gu J.N., Liang J.X., Wang L.J., Xue Y.X., Li K., Guo M.M., Sun T.H., Jia J.P. (2025). Suppressed lattice oxygen mobility on Ag/FeO_x_ catalyst enhances the sulfur selectivity of H_2_S selective oxidation. J. Hazard. Mater..

[12] Shah M.S., Tsapatsis M., Siepmann J.I. (2017). Hydrogen sulfide capture: From absorption in polar liquids to oxide, zeolite, and metal–organic framework adsorbents and membranes. Chem. Rev..

[13] Gupta N.K., Bae J., Kim K.S. (2021). A novel one-step synthesis of Ce/Mn/Fe mixed metal oxide nanocomposites for oxidative removal of hydrogen sulfide at room temperature. RSC Adv..

[14] Meng F.N., Di X.P., Dong H.W., Zhang Y., Zhu C.L., Li C.Y., Chen Y.J. (2013). Ppb H_2_S gas sensing characteristics of Cu_2_O/CuO sub-microspheres at low-temperature. Sens. Actuators.

[15] Lin Y.T., Li Y.R., Wang B., Tian J.L., Liu H.Q., Li Y.R., Xu Z.C., Cao Q., Zhu T.Y. (2025). Pilot-scale testing on catalytic hydrolysis of carbonyl sulfur combined with absorption-oxidation of H_2_S for blast furnace gas purification. J. Environ. Sci..

[16] Xiong Y.R., Wang L.L., Ning P., Luo J.F., Li X., Yuan L., Xie Y.B., Ma Y.X., Wang X.Q. (2024). Constructing oxygen vacancy-enriched Fe_3_O_4_@MnO_2_ core-shell nanoplates for highly efficient catalytic oxidation of H_2_S in blast furnace gas. Sep. Purif. Technol..

[17] Cao E.P., Zheng Y.H., Zhang H., Wang J.S., Li Y.R., Zhu T.Y., Zhang Z.G., Xu G.W., Cui Y.B. (2024). In-situ regenerable Cu/Zeolite adsorbent with excellent H_2_S adsorption capacity for blast furnace gas. Sep. Purif. Technol..

[18] Permatasari P., Hendrik G.P., Sholihah F., Jibran M. Chemical scrubbing for removal of carbon dioxide and hydrogen sulfide in biogas purification process. Proceedings of the 6th International Conference on Applied Engineering, ICAE.

[19] Tarek M., Santos J.S., Márquez V., Fereidooni M., Yazdanpanah M., Praserthdam S., Praserthdam P. (2024). A critical review towards the causes of the iron-based catalysts deactivation mechanisms in the selective oxidation of hydrogen sulfide to elemental sulfur from biogas. J. Energy Chem..

[20] Jirasansawat K., Chiemchaisri W., Chiemchaisri C. (2024). Enhancement of sulfide removal and sulfur recovery in piggery wastewater via lighting-anaerobic digestion with bioaugmentation of phototrophic green sulfur bacteria. Environ. Sci. Pollut. Res..

[21] Bhatt P.M., Belmabkhout Y., Assen A.H., Weseliński Ł.J., Jiang H., Cadiau A., Xue D.X., Eddaoudi M. (2017). Isoreticular rare earth fcu-MOFs for the selective removal of H_2_S from CO_2_ containing gases. Chem. Eng. J..

[22] Ren M.L., Fan F.C., Zhou B., Liang X.Y., Yang Z. (2022). Dynamic simulation of adsorption desulfurization from diesel fuel over activated carbon in the fixed bed. Chem. Eng. Res. Des..

[23] Paz L., Gentil S., Fierro V., Celzard A. (2025). Activated carbons outperform other sorbents for biogas desulfurization. Chem. Eng. J..

[24] Sun R.J. (2020). Research on New Technology of Blast Furnace Gas Desulfurization. Master’s Thesis.

[25] Min G.H., Park H.J., Bhatti U.H., Jang J.T., Baek I.H., Nam S.C. (2023). Hydrogen sulfide removal from low concentration gas streams using metal supported mesoporous silica SBA-15 adsorbent. Microporous Mesoporous Mater..

[26] Watanabe S. (2021). Chemistry of H_2_S over the surface of common solid sorbents in industrial natural gas desulfurization. Catal. Today.

[27] Yang C., Yang S., Fan H.L., Wang J., Wang H., Shangguan J. (2020). A sustainable design of ZnO-based adsorbent for robust H_2_S uptake and secondary utilization as hydrogenation catalyst. Chem. Eng. J..

[28] Guo Z.C. (2024). Basic Research on Microcrystalline Materials and Phase Transfer Catalysts Used to Crude Benzene Desulfurization. Master’s Thesis.

[29] Kanca A., Alpsoy Z., Ata O.N. (2023). Sulfidation performance of unsupported and SBA 15-supported Ca-based mixed metal oxides. Int. J. Hydrogen Energy.

[30] Zhan Y.Y., Shen L.J., Xu C.B., Zhao W.T., Cao Y.N., Jiang L.L. (2018). MOF-derived porous Fe_2_O_3_ with controllable shapes and improved catalytic activities in H_2_S selective oxidation. Crystengcomm.

[31] Liu X., Zhai X.X., Zhao Y.H., Shan L., Liu Z.Q., Liu Y.F. (2025). Sulfur modified N-doped carbocatalysts promote the selectivity for H_2_S selective oxidation. Appl. Catal. B.

[32] Yashina L.V., Zyubin A.S., Püttner R., Zyubina T.S., Neudachina V.S., Stojanov P., Riley J., Dedyulin S.N., Brzhezinskaya M.M., Shtanov V.I. (2011). The oxidation of the PbS (001) surface with O_2_ and air studied with photoelectron spectroscopy and ab initio modeling. Surf. Sci..

[33] Umek P., Gloter A., Pregelj M., Dominko R., Jagodic M., Jaglicic Z., Zimina A., Brzhezinskaya M., Potocnik A., Filipic C. (2009). Synthesis of 3D hierarchical self-assembled microstructures formed from α-MnO_2_ nanotubes and their conducting and magnetic properties. J. Phys. Chem. C.

[34] Wu M.M., Su Z.B., Fan H.L., Mi J. (2017). New way of removing hydrogen sulfide at a high temperature: Microwave desulfurization using an iron-based sorbent supported on active coke. Energy Fuel.

[35] Rezaei S., Jarligo M.O.D., Wu L., Kuznicki S.M. (2015). Breakthrough performances of metal-exchanged nanotitanate ETS-2 adsorbents for room temperature desulfurization. Chem. Eng. Sci..

[36] Oh W.D., Lei J.X., Veksha A., Giannis A., Lisak G., Chang V.W.C., Hu X., Lim T.T. (2018). Influence of surface morphology on the performance of nanostructured ZnO-loaded ceramic honeycomb for syngas desulfurization. Fuel.

[37] Feng Y., Shi L., Zhang S.S., Wu M.M., Mi J. (2017). Kinetics study of zinc oxide sorbent prepared by different methods for hot coal gas desulfurization. Chem. Ind. Eng. Prog..

[38] Wang J., Guo J., Parnas R., Liang B. (2015). Calcium-based regenerable sorbents for high temperature H_2_S removal. Fuel.

[39] Li H.F., Su S., Hu S., Xu K., Jiang L., Wang Y., Xu J., Xiang J. (2018). Effect of preparation conditions on Mn_x_O_y_/Al_2_O_3_ sorbent for H_2_S removal from high-temperature synthesis gas. Fuel.

[40] Cimino S., Lisi L., Falco G.D., Montagnaro F., Balsamo M., Erto A. (2018). Highlighting the effect of the support during H_2_S adsorption at low temperature over composite Zn-Cu sorbents. Fuel.

[41] Sánchez-Hervás J.M., Maroño M., Fernández-Martínez R., Ortiz I., Ortiz R., Gómez-Mancebo M.B. (2022). Novel ZnO-NiO-graphene-based sorbents for removal of hydrogen sulfide at intermediate temperature. Fuel.

[42] Kim S., Gupta N.K., Bae J., Kim K.S. (2021). Fabrication of coral-like Mn_2_O_3_/Fe_2_O_3_ nanocomposite for room temperature removal of hydrogen sulfide. J. Environ. Chem. Eng..

[43] Xuan Y.N., Yu Q.B., Qin Q., Wang K., Duan W.J., Liu K.J., Zhang P. (2018). Selection of desulfurizer and control of reaction products on flue-gas desulfurization using chemical-looping technology. Energy Fuel.

[44] Xuan Y.N., Yu Q.B., Gao H.T., Wang K., Duan W.J. (2020). Modular manganese/diatomite-Santa Barbara Amorphous-15 sorbent for moderate-temperature flue gas desulfurization. Chem. Eng. J..

[45] Li H.F. (2020). The Experimental Research and Mechanism on Composite Sorbent for H_2_S Removal from Coal Gas. Ph.D. Thesis.

[46] Ko T.H., Chu H., Lin H.P., Peng C.Y. (2006). Red soil as a regenerable sorbent for high temperature removal of hydrogen sulfide from coal gas. J. Hazard. Mater..

[47] Ko T.H., Chu H., Tseng J.J. (2006). Feasibility study on high-temperature sorption of hydrogen sulfide by natural soils. Chemosphere.

[48] Subsadsana M., Kham-or P., Sangdara P., Suwannasom P., Ruangviriyachai C. (2017). Synthesis and catalytic performance of bimetallic NiMo-and NiW-ZSM-5/MCM-41 composites for production of liquid biofuels. J. Fuel Chem. Technol..

[49] Chang S., Ren G.X., Gui Y.H., Li Y.M., Wang Z.D. (2023). Thermodynamic Analysis of 316L Embedded Chromium Infiltration Process Based on HSC-Chemistry. Metal Mater. Metal. Eng..

[50] Wei J., Deng Q., Liu X.M., Gu X.F., Wang W.Z. (2017). Application of HSC chemistry software in the determination of chemical thermodynamic function by measuring electromotance. Guangdong Chem. Ind..

[51] Westmoreland P.R., Harrison D.P. (1976). Evaluation of candidate solids for high-temperature desulfurization of low-Btu gases. Environ. Sci. Technol..

[52] Abdalla A., Farooqui A., Mohamedali M., Mahinpey N. (2024). Copper-based chemical looping air separation process: Thermo-dynamics, kinetic modeling, and simulation of the fluidized beds. Sep. Purif. Technol..

[53] López-Ortiz A., González-Vargas P.E., Meléndez-Zaragoza M.J., Collins-Martínez V. (2017). Thermodynamic analysis and process simulation of syngas production from methane using CoWO4 as oxygen carrier. Int. J. Hydrogen Energy.

[54] Jerndal E., Mattisson T., Lyngfelt A. (2006). Thermal analysis of chemical-looping combustion. Chem. Eng. Res. Des..

[55] Wang Y.K. (2013). Application of HSC chemistry software in university chemical scientific research. J. Henan Inst. Edu..

[56] Du J.F., Wu G.H., Feng X.H., Jin H., Huang F., Wang H.B. (2024). Research on fine desulfurization process route of blast furnace gas in steel industry. Ind. Furn..

[57] Xia H., Chang X.Q., Liu B.S. (2017). High-temperature H_2_S removal performance over ordered mesoporous La-Mn-supported Al_2_O_3_-CaO sorbents. Chem. Eng. J..

[58] Mi J., Zhang Y.Y., Zhu Y.S., Guo T., Fan H.L. (2011). Semi-coke-supported mixed metal oxides for hydrogen sulfide removal at high temperatures. Environ. Eng. Sci..

[59] Li T., Ren X.R., Bao L.X., Wang M.J., Bao W.R., Chang L.P. (2020). Effect of lignite as support precursor on deep desulfurization performance of semicoke supported zinc oxide sorbent in hot coal gas. RSC Adv..

[60] Garces H.F., Galindo H.M., Garces L.J., Hunt J., Morey A., Suib S.L. (2010). Low temperature H_2_S dry-desulfurization with zinc oxide. Microporous Mesoporous Mater..

[61] Guo L.F., Pan K.L., Lee H.M., Chang M.B. (2015). High-temperature gaseous H_2_S removal by Zn–Mn-based sorbent. Ind. Eng. Chem. Res..

[62] Li R., Krcha M.D., Janik M.J., Roy A.D., Dooley K.M. (2012). Ce-Mn oxides for high-temperature gasifier effluent desulfurization. Energy Fuel.

[63] Vamvuka D., Arvanitidis C., Zachariadis D. (2004). Flue gas desulfurization at high temperatures: A Review. Environ. Eng. Sci..

[64] Fang H.B., Zhao J.T., Fang Y.T., Huang J.J., Wang Y. (2013). Selective oxidation of hydrogen sulfide to sulfur over activated carbon-supported metal oxides. Fuel.

[65] Wang J., Wang L.L., Fan H.L., Wang H., Hu Y.F., Wang Z.D. (2017). Highly porous copper oxide sorbent for H_2_S capture at ambient temperature. Fuel.

[66] Cristiano D.M., Mohedano R.D.A., Nadaleti W.C., Junior A.B.D.C., Lourenço V.A., Gonçalves D.F.H., Filho P.B. (2020). H_2_S adsorption on nanostructured iron oxide at room temperature for biogas purification: Application of renewable energy. Renew. Energy.

[67] (2025). Price of Metal Powder, *China Power Network*. www.cnpowder.com.cn.

[68] Zhao R.Z., Gao G.P., Wang K., Chen X.Q., Ji W.Q. (2024). Current situation and tendency of blast furnace gas fine desulferization technology in iron and steel industry. Environ. Sci. Manag..

[69] Hasegawa Y.I., Maki R.U., Sano M., Miyake T. (2009). Preferential oxidation of CO on copper-containing manganese oxides. Appl. Catal. A-Gen..

[70] Hu H., Wang S.X., Zhang X.L., Zhao Q.Z., Li J. (2006). Study on simultaneous catalytic reduction of sulfur dioxide and nitric oxide on rare earth mixed compounds. J. Rare Earths.

[71] André L., Abanades S., Cassayre L. (2017). High-temperature thermochemical energy storage based on redox reactions using Co-Fe and Mn-Fe mixed metal oxides. J. Solid State Chem..

[72] Chanapattharapol K.C., Krachuamram S., Youngme S. (2017). Study of CO_2_ adsorption on iron oxide doped MCM-41. Microporous Mesoporous Mater..

[73] Hiremath V., Shavi R., Seo J.G. (2017). Controlled oxidation state of Ti in MgO-TiO_2_ composite for CO_2_ capture. Chem. Eng. J..

[74] Kariya J., Ryu J., Kato Y. (2016). Development of thermal storage material using vermiculite and calcium hydroxide. Appl. Therm. Eng..

[75] Darband G.B., Aliofkhazraei M., Hamghalam P., Valizade N. (2017). Plasma electrolytic oxidation of magnesium and its alloys: Mechanism, properties and applications. J. Magn. Alloy.

[76] Wang X.F., Liu Y.Y., Ge W., Xu Y., Jia H.L., Li Q.B. (2023). Complete oxidation of lean methane over metal oxide supported Pd catalysts: Current advancement and future perspectives. J. Environ. Chem. Eng..

[77] Alalwan H.A., Alminshid A.H., Mohammed M.M., Mohammed M.F. (2023). Methane activation on metal oxide nanoparticles: Spectroscopic identification of reaction mechanism. Part. Sci. Technol..

[78] Zhang X.L. (2020). Synthesis of Manganese Based Catalyst for Desulfurization and Denitrification from Low-Temperature Flue Gas. Master’s Thesis.

[79] Maroño M., Ortiz I., Sánchez J.M., Alcaraz L., Alguacil F.J., López F.A. (2021). Effective removal of hydrogen sulfide using Mn-based recovered oxides from recycled batteries. Chem. Eng. J..

[80] Li J., Chang J.C., Ma C.Y., Feng T., Zhang L.Q., Wang T., Song Z.L. (2025). Innovative research on one-step regeneration and reduction of saturated desulfurization coke: Reactivating desulfurization performance and sulfur recovery. Fuel.

[81] Lee J., Ahn Y., Cho H., Kim J. (2022). Economic performance assessment of elemental sulfur recovery with carbonate melt desulfurization process. Process. Saf. Environ..

[82] Song J.X., Liu M.Y., Ma X.C., Tian Q.W., Feng J.K., Zhong X.T., Duan F. (2023). Thermal decomposition behavior and computational analysis of alpha and beta manganese dioxide nanorods. J. Alloys Compd..

[83] Li L., He M.Z., Zhang A.H., Zhou J. (2011). A study on non-isothermal kinetics of the thermal decompositions of β-manganese dioxide. Thermochim. Acta.

[84] Dearden B.R., Edwards A.C., Evans Z.T., Woolsey B., Blair C.R., Harrison N.G., Harrison R.G. (2022). Synthesis of zinc oxide nanoplates and their use for hydrogen sulfide adsorption. J. Sol-Gel Sci. Technol..

[85] Hu J.W., Poelman H., Theofanidis S.A., Joos J.J., Detavernier C., Poelman D., Wei W., Galvita V.V. (2023). High temperature H_2_S removal via CO_2_-assisted chemical looping over ZrO_2_-modified Fe_2_O_3_. Appl. Catal. B.

[86] Li Y.K., Yang C., Fan H.L., Wang Y.S., Duan M.X., Feng Y.T., Lin J.Y. (2022). Enhanced sulfur selectivity for H_2_S catalytic oxidation over Fe_2_O_3_@UiO-66 catalyst. Sep. Purif. Technol..

[87] Long N., Loc T. (2016). Experimental and modeling study on room-temperature removal of hydrogen sulfide using a low-cost extruded Fe_2_O_3_-based adsorbent. Adsorption.

[88] Wang Y.J., Liao J.J., Chang L.P., Bao W.R., Ma J.H. (2025). Research progress of the fine desulfurization technology for blast furnace gas. Mod. Chem. Ind..

[89] Du S., Liu X., Liu Y., Wang J.H., Liu D.X., Yang J.X., Zhang X. (2023). Bamboo derived activated carbon as a highly efficient catalyst for the oxidation and adsorption of hydrogen sulfide at room temperature. Environ. Sci. Nano.

[90] Azamuddin M.F.A., Abdullah N., Nor N.M. (2021). Physicochemical characteristics of activated carbon impregnated with different type of metal oxide nanoparticles towards hydrogen sulfide removal. IOP Conf. Ser. Earth Environ. Sci..

[91] Sun D., Yang L., Liu N., Jiang W.J., Jiang X., Li J.J., Yang Z.Y., Song Z.P. (2020). Sulfur resource recovery based on electrolytic manganese residue calcination and manganese oxide ore desulfurization for the clean production of electrolytic manganese. Chin. J. Chem. Eng..

[92] Alsehli B.R. (2023). Toward sustainable environmental cleanup: Metal–organic frameworks in adsorption-a review. Desalination Water Treat..

[93] Li J.N., Yuan Y.B., Zhang J.D., Li N., Guo Q., Yu Y.Y., Huang Q.Q., Wei X.P., Jiang J. (2025). Activation of oxygen by manganese sulfide to produce reactive oxygen: Species, kinetics and reaction mechanisms. J. Civil Environ. Eng..

[94] Song Y., Zhang H.N., Ren L. (2024). A review of research on MnS inclusions in high-quality steel. Eng. Rep..

[95] Wadhawan A.R., Livi K.J., Stone A.T., Bouwer E.J. (2015). Influence of oxygenation on chromium redox reactions with manganese sulfide (MnS(s)). Environ. Sci. Technol..

[96] Gorynski C., Geiß J., Anselmi-Tamburini U., Winterer M. (2024). Structural and compositional gradients in alternating current sintered aluminum-doped zinc oxide. Acta Mater..

[97] Gnanasagaran C.L., Ramachandran K., Ramesh S., Ubenthiran S., Jamadon N.H. (2023). Effect of co-doping manganese oxide and titania on sintering behaviour and mechanical properties of alumina. Ceram. Int..

